# Supplementary material for: CRISPR-Cas systems are widespread accessory elements across bacterial and archaeal plasmids
Source: Nucleic Acids Res. 2021 Oct 4;50(8):4315–28. doi: 10.1093/nar/gkab859 (PMC9071438; doi:10.1093/nar/gkab859)
Supplement: gkab859_Supplemental_Files [file gkab859_supplemental_files.zip › Supplementary Material.pdf]

SUPPLEMENTARY MATERIAL

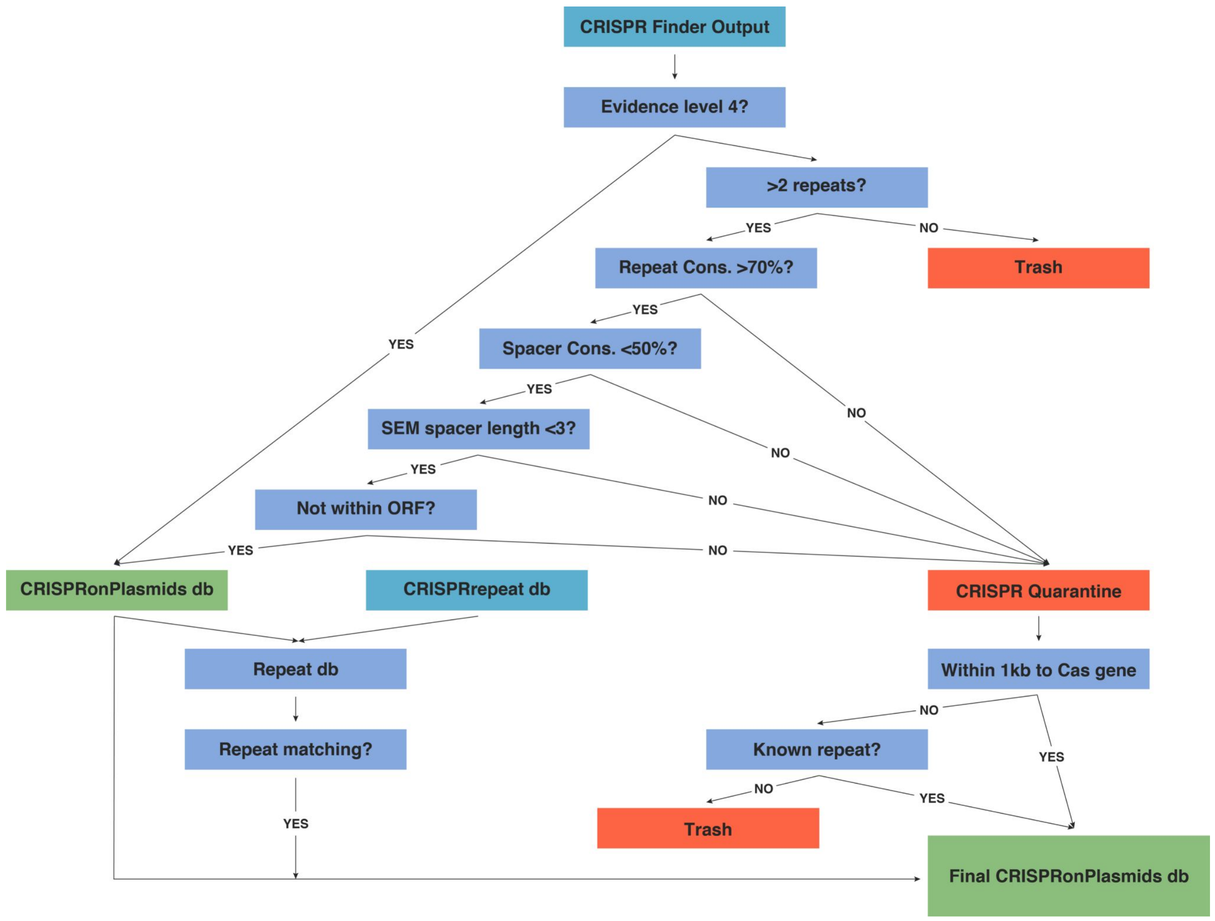

Supplementary Figure S1. Decision tree used for elimination of false-positive arrays from the outputs of CRISPRCasFinder and inclusion of undetected CRISPRs. Briefly, high confidence arrays (evidence level 4) predicted by CRISPRCasFinder were automatically retained and included in the final CRISPROnPlasmids database (db). Arrays with lower evidence level scores were removed if the number of repeats was less than three; arrays with more than two repeats were placed into a quarantine list if the calculated average repeat conservation (cons.) across the array was higher than 70%, the spacer conservation was lower than 50%, the standard error of the mean (SEM) of the spacer lengths was less than 3, and if the array did not overlap with a predicted high confidence open reading frame (ORF). Putative arrays from the quarantine arrays were subsequently rescued for the analyses if they were located within 1 Kb of a predicted *cas* gene or if they matched (>95% coverage and identity) with high confidence CRISPR repeats (known repeats). Finally, the repeat db was blasted against plasmid/chromosome sequences to identify arrays that had been missed by the previous algorithm (repeat matching). Hits were considered true arrays when more than three repeats were identified (>95% identity and coverage), each within <100 bp from each other.

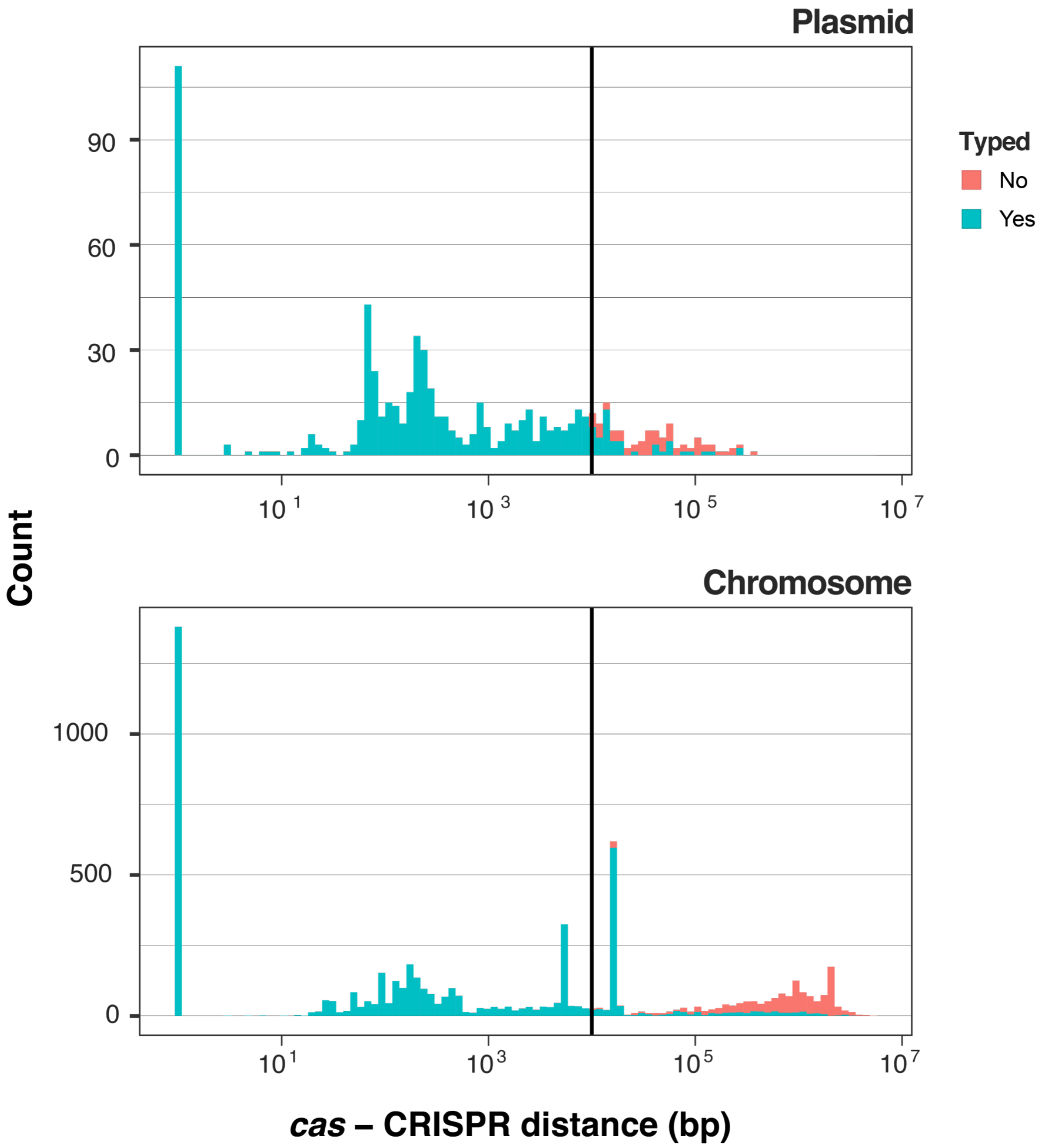

Supplementary Figure S2. Distances between CRISPR arrays and the nearest *cas* operon. Only de-replicated plasmids and associated host chromosomes are included. All distances above 100 kb were grouped together in the 100 kb mark. Color denotes whether CRISPR arrays could be subtyped (blue; true) either by proximity to a *cas* operon (<10 kb threshold) or by repeat similarity with an array proximal to a *cas* operon (> 85% identity). Unassigned arrays are marked in red (false). Note that this does not include CRISPR arrays on contigs without any *cas* operon, which are the largest share of truly orphan arrays.

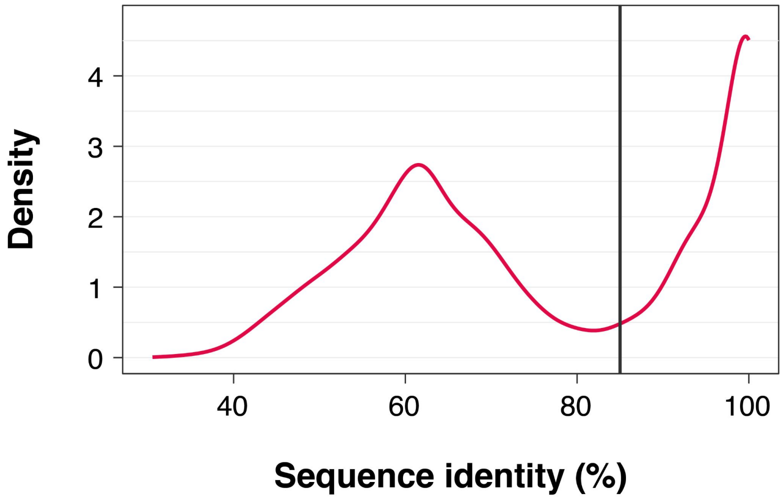

Supplementary Figure S3. Density plot of sequence identities between consensus repeats of CRISPR arrays originating from the same contig. Vertical line shows the chosen cutoff for typing “distant CRISPR arrays”: orphan arrays that could be associated to a CRISPR-Cas locus within the same contig/genome.

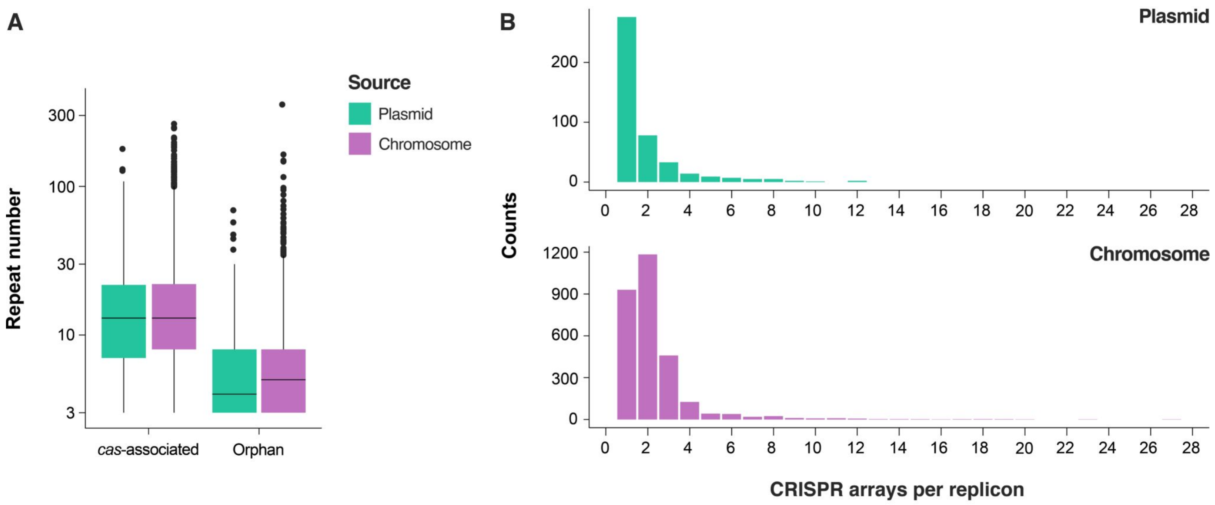

Supplementary Figure S4. (A) Average array lengths of orphan and *cas*-associated CRISPRs originating from plasmids (green) and plasmid-host chromosomes (purple). Orphan CRISPRs have significantly fewer repeats ($p<2e-16$, negative-binomial GLM), but in each group there is no difference between chromosomal and plasmid CRISPR lengths ($p=0.248$), and no interaction between the two ($p=0.134$) **(B)** Frequency distribution of the number of CRISPR arrays per plasmid and plasmid-host chromosomes. Only replicons with at least 1 array are displayed.

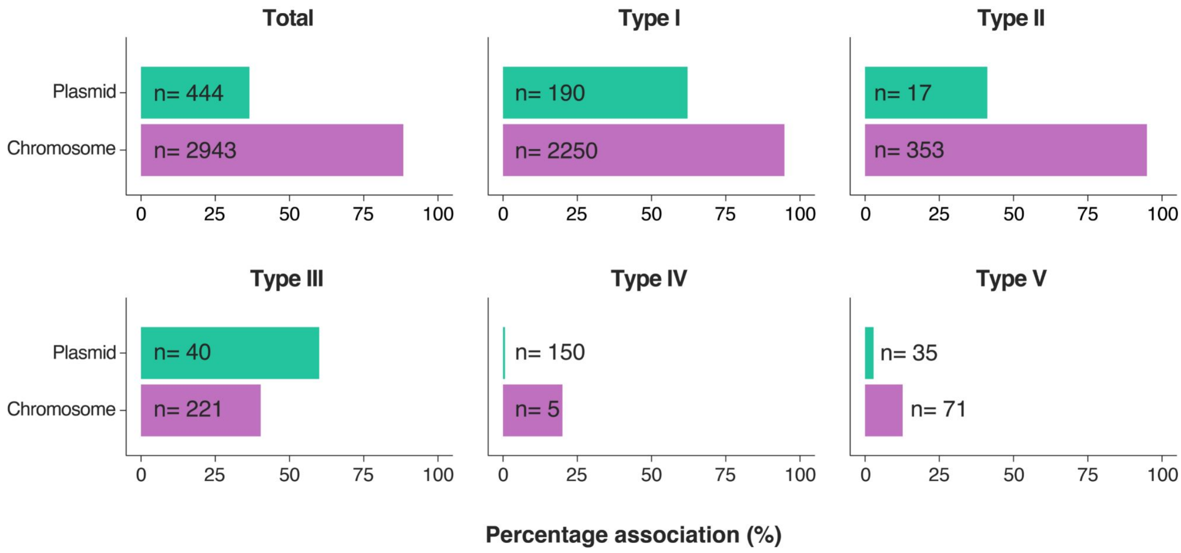

Supplementary Figure S5. Association of adaptation modules with CRISPR-Cas systems encoded by plasmids and plasmid-host chromosomes. Percentage of CRISPR-Cas systems, separated at the type level, predicted to encode adaptation components (Cas1 and/or Cas2). The aggregate value (averaging data from all CRISPR-Cas types) is shown under “Total”.

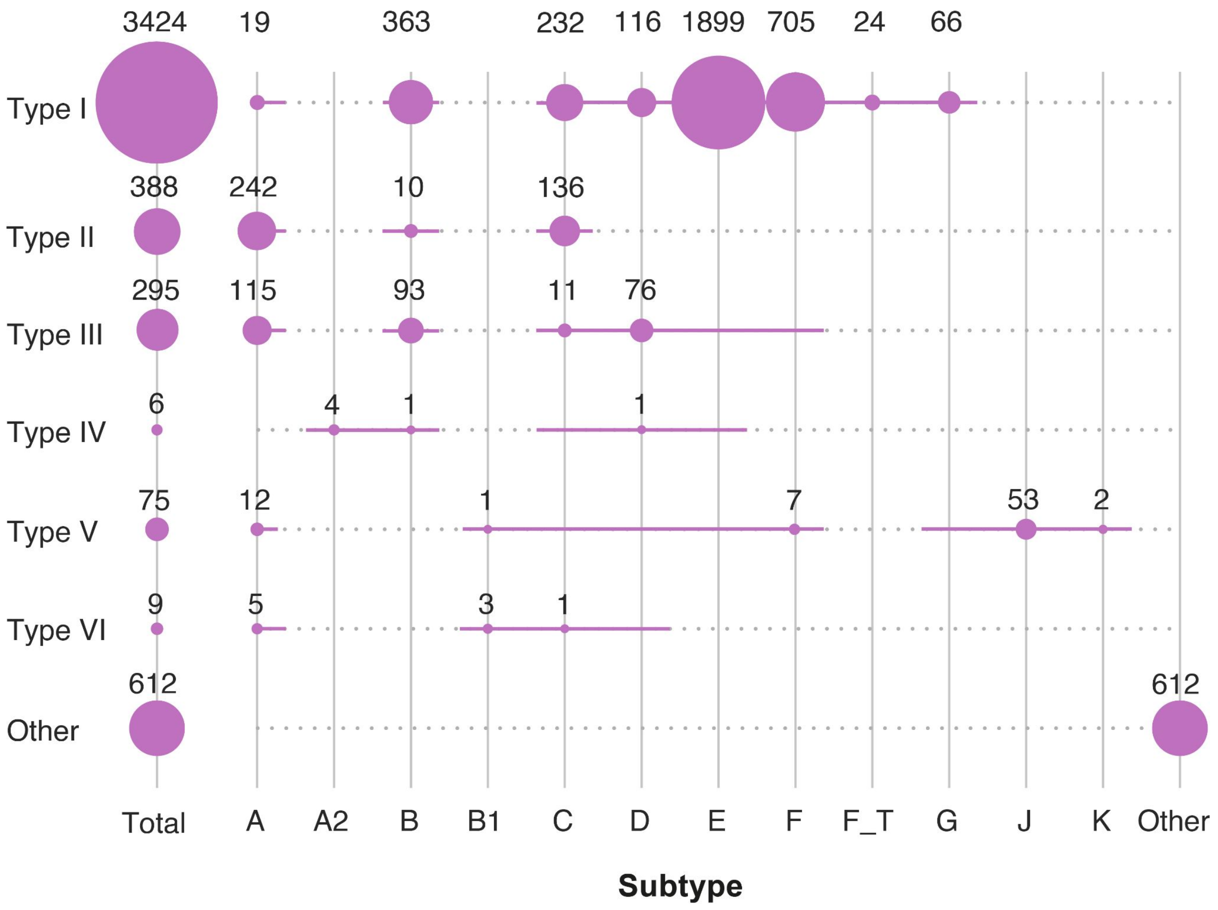

Supplementary Figure S6. Distribution and prevalence of CRISPR-Cas subtypes across plasmid-associated host chromosomes. "Other" represents systems that could not be unambiguously assigned (e.g. multiple equally scoring subtypes, co-localised/hybrid systems, and orphan-untyped components). "I-F_T" refers to the transposon-associated subtype I-F variant and subtype IV-A is subdivided into its known variants (IV-A1 to A3). Total counts per CRISPR-Cas type are summarised on the left. The horizontal discontinuous line (instead of continuous purple) indicates that the particular subtypes are not described in the current CRISPR-Cas classification.

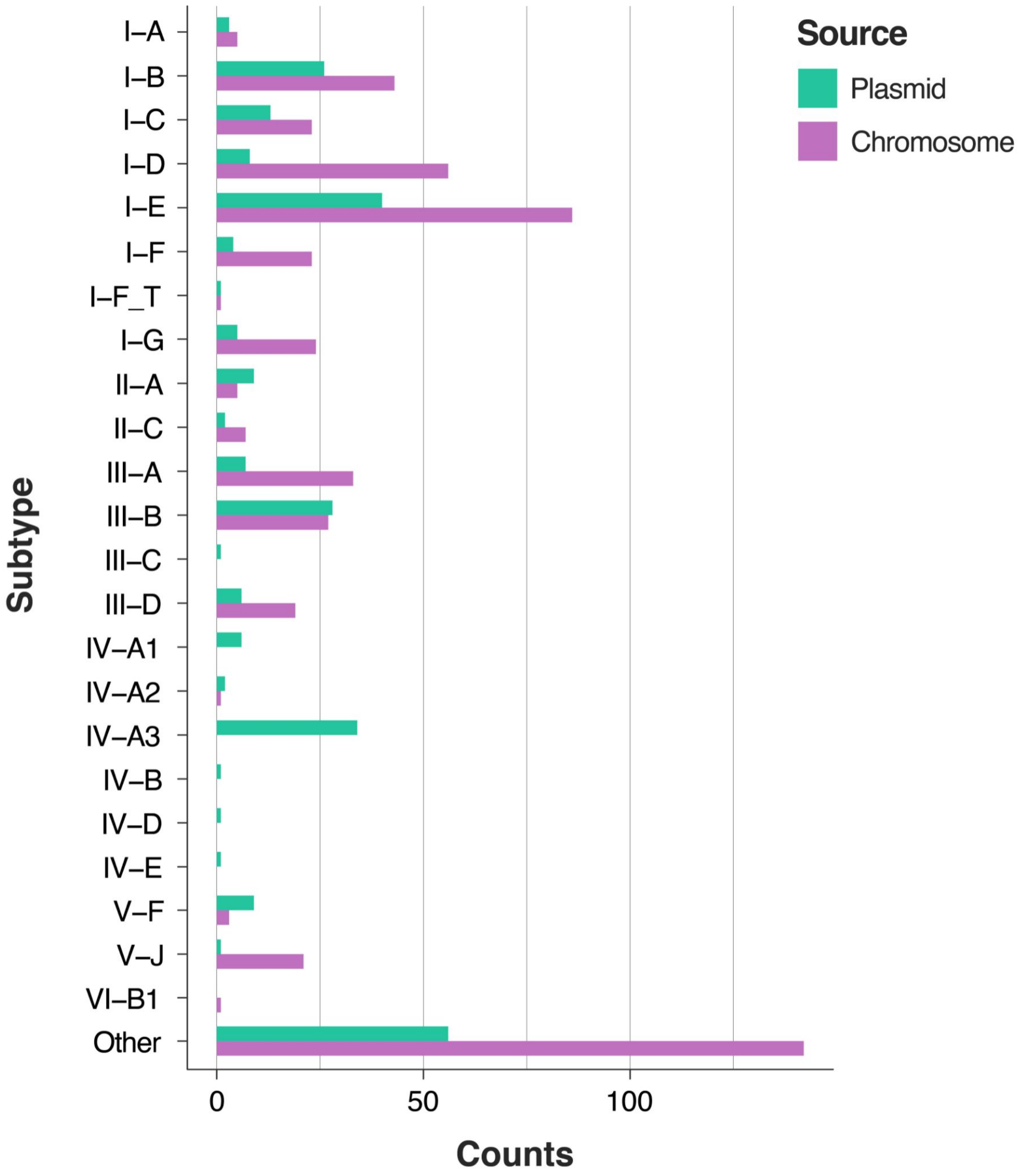

Supplementary Figure S7. Direct comparison of the distribution and prevalence of CRISPR-Cas subtypes across plasmids and their plasmids-associated chromosomes, where the plasmid and chromosome each carry at least one CRISPR-Cas. Number of plasmid- and chromosomal systems (green and purple, respectively) are shown, broken down at the subtype level (or variant level, when possible).

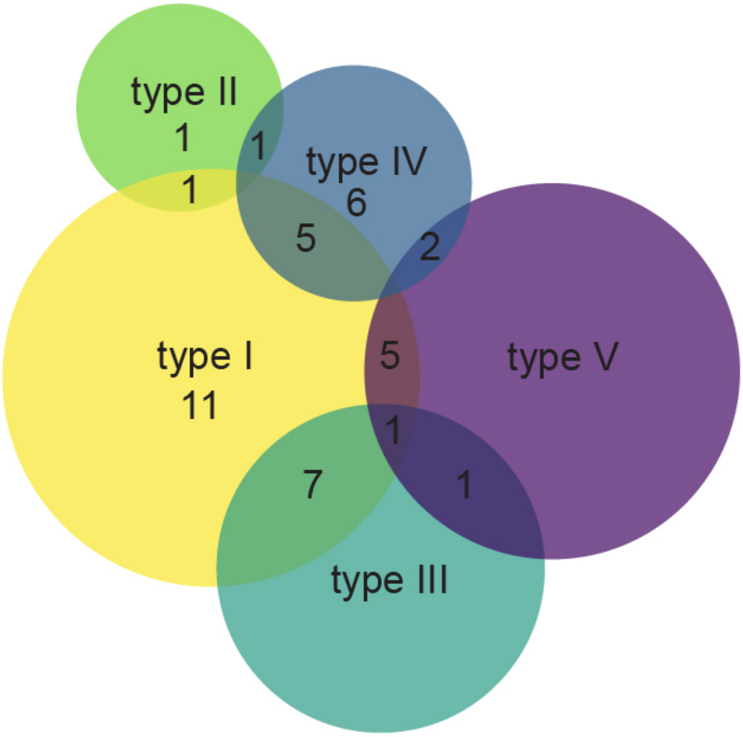

Supplementary Figure S8: Co-occurrence of multiple CRISPR-Cas types on individual plasmids. The different CRISPR-Cas systems are color coded according to their type classification: I in yellow, II in light green, III in teal, IV in dark blue, and V in purple. The overlapping areas in the Venn diagram indicate co-occurrences of the different CRISPR-Cas types, where the number (n) of observations is indicated. For plasmids encoding type I systems, co-occurrence of different subtypes were also detected and the number of observations are indicated (n = 11).

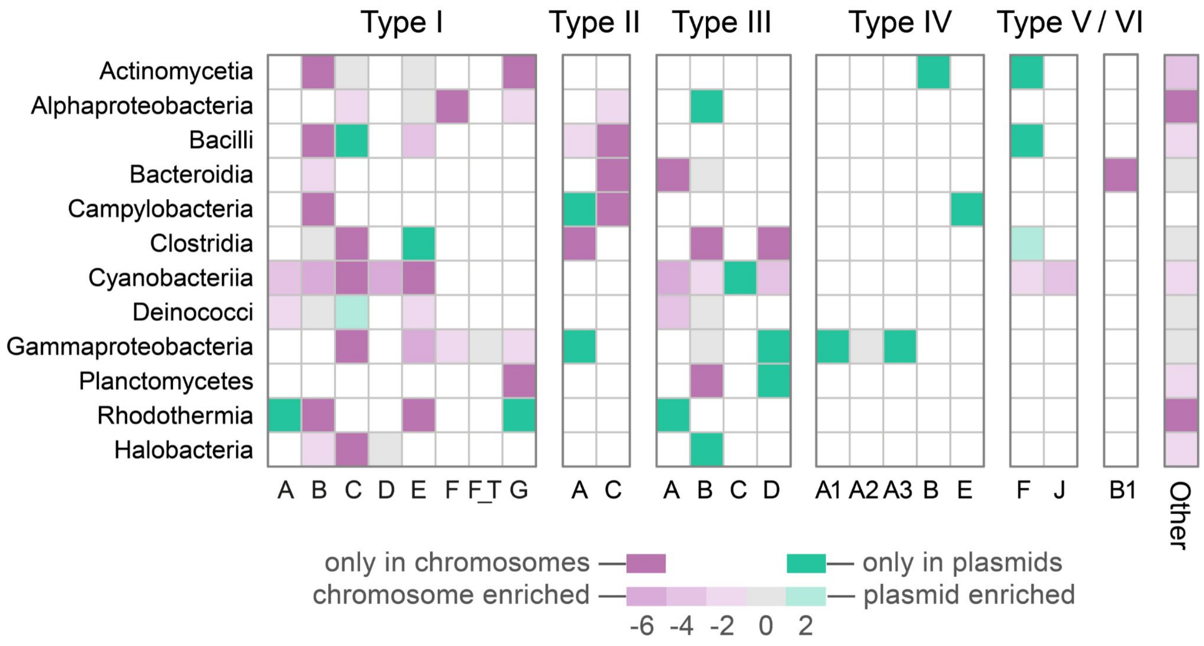

Supplementary Figure S9. Direct comparison between CRISPR-Cas subtype prevalence across plasmids and their host-chromosomes (per taxa). Only plasmid-host chromosome pairs for which the plasmid and chromosome each contain at least one CRISPR-Cas are included in this analysis. Blank spaces represent subtypes for which no data is available. Color gradient denotes the log2 ratio between prevalence on plasmids and prevalence on chromosomes, such that positive values indicate plasmid enrichment and negative values indicate chromosome enrichment. The darkest shades of green and purple indicate subtypes only present in plasmids and chromosomes, respectively.

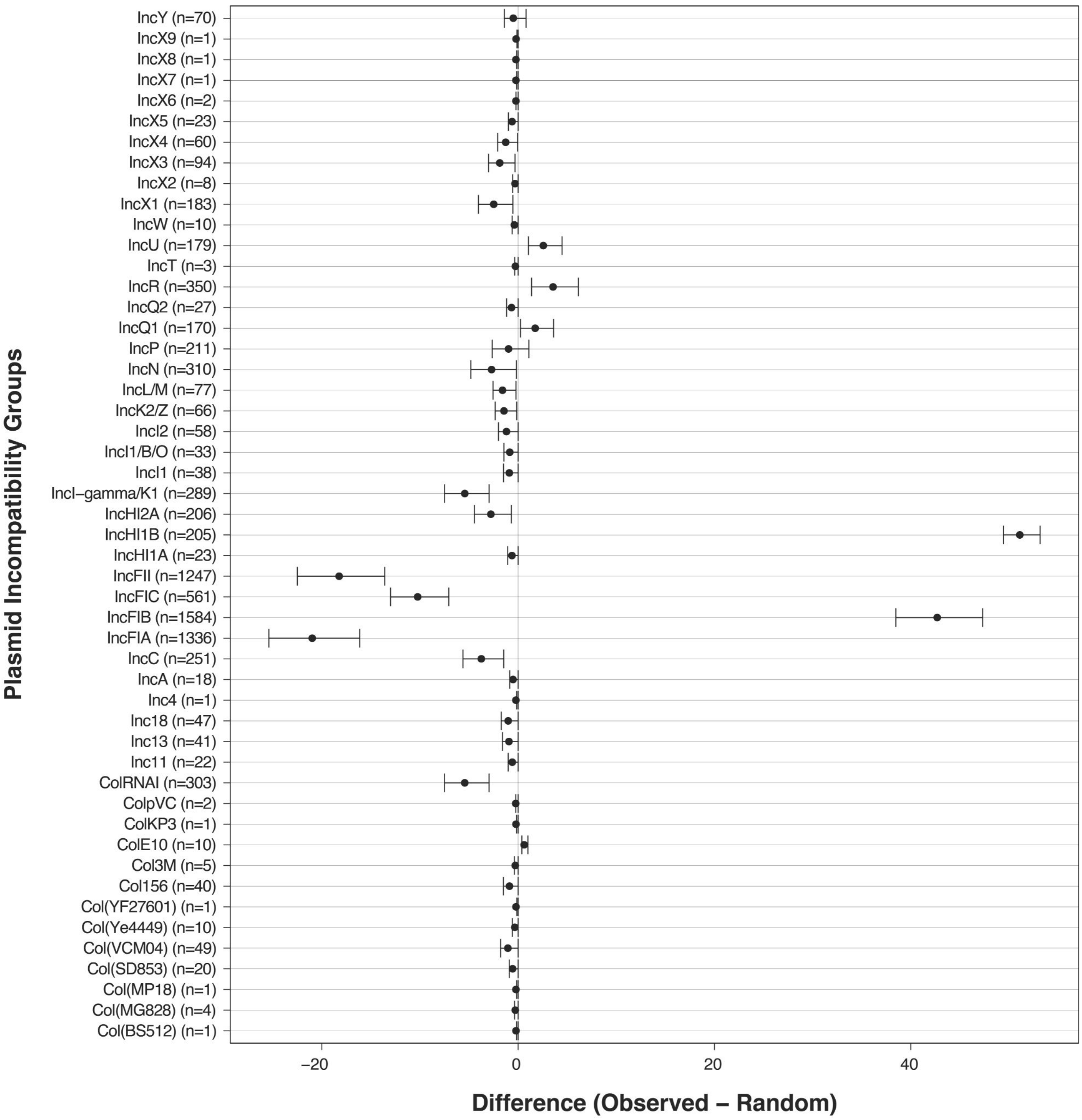

Supplementary Figure S10. Distribution of CRISPR-Cas systems across plasmid Incompatibility (Inc) groups. Enrichment of CRISPR-Cas loci within plasmid incompatibility groups within the Inc-typeable fraction of the complete plasmid dataset. Single plasmids can belong to more than one Inc group. The observed distribution of CRISPR-Cas on Inc-typable plasmids was compared with a random distribution, in which CRISPR-Cas systems were placed in random Inc-typable plasmids. The mean and standard deviation from 1000 random permutations is shown. The difference is the number of observed CRISPR-Cas systems subtracted by the number of CRISPR-Cas systems in the random permutation.

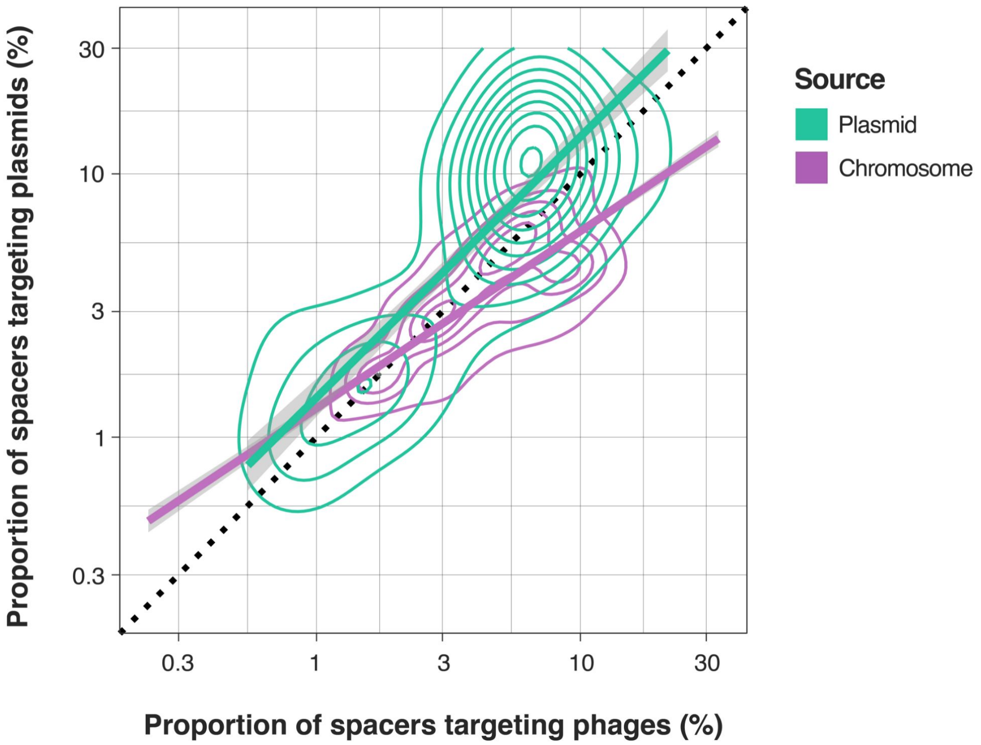

Supplementary Figure S11. Proportion of spacers matching viral/plasmid sequences across all detected CRISPR arrays. Contour plot depicting the global targeting preference (plasmid vs. virus) for spacers within single arrays derived from plasmids (green) and plasmid-host chromosomes (purple). Regression lines with shaded areas correspond to 95% confidence intervals. The black dotted line indicates a 1:1 proportion of virus- vs plasmid-targeting spacers.

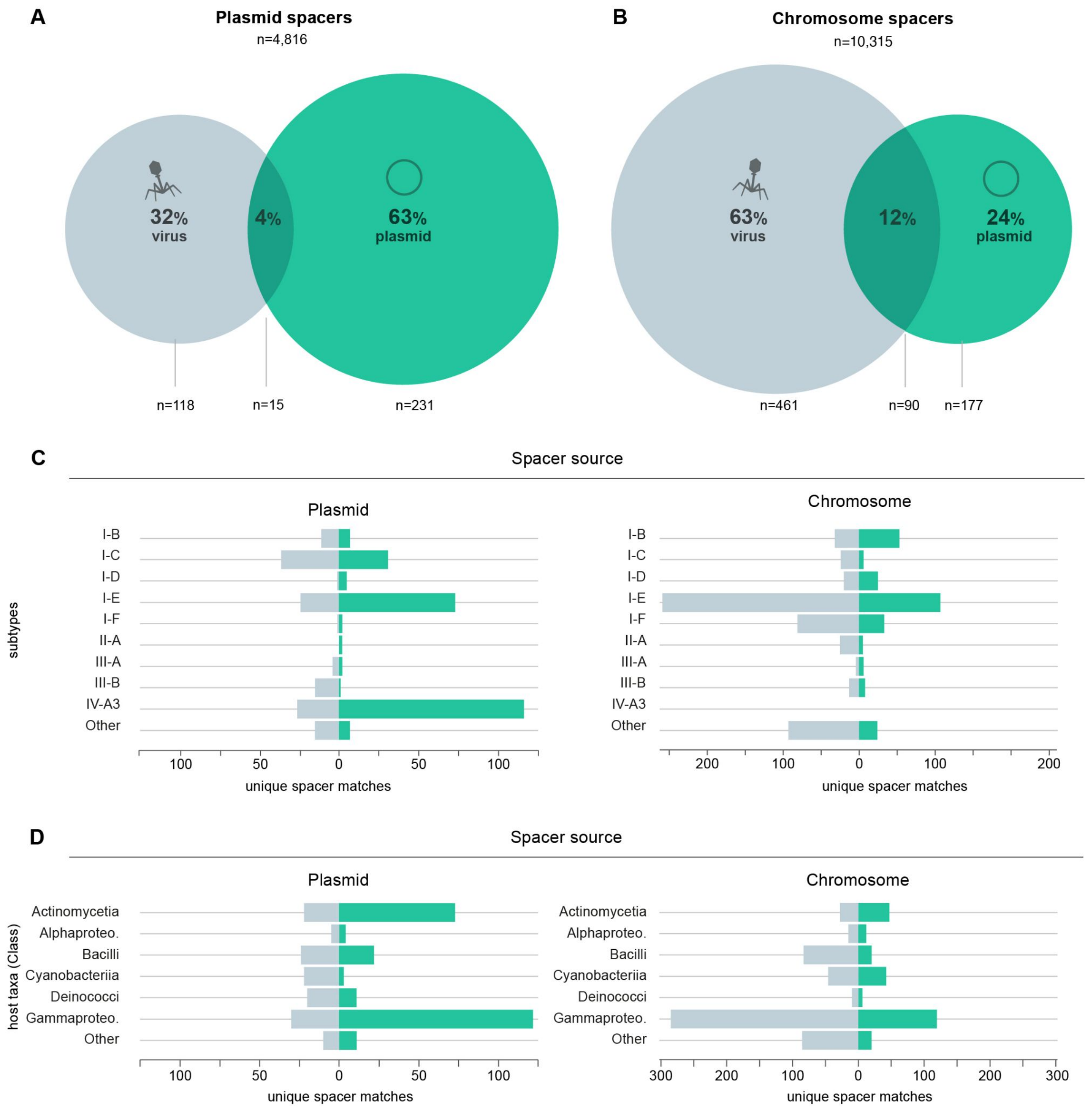

Supplementary Figure S12. Direct comparison plasmid-host: only cells where both chromosome and (at least one) plasmid has CRISPR. The proportion of plasmid (**A**) and plasmid-host (**B**) chromosomal spacers matching plasmids (green) and viruses (grey). **(C)** Distribution of spacer-protospacer matches derived from plasmid (left) and plasmid-host chromosome (right) spacer contents, presented according to CRISPR-Cas subtype/variant and predicted spacer target: plasmids (green) and viruses (grey). Only the top 9 subtypes for both plasmids and chromosomes are represented, with the remaining grouped in “Other”. **(D)** Distribution of spacer-protospacer matches derived from plasmid (left) and host chromosome (right) spacer contents, broken down by host taxa and predicted protospacer origin: plasmids (green) and viruses (grey). Only the top 6 classes for both plasmids and chromosomes are represented, with the remaining classes grouped in “Other”.

A

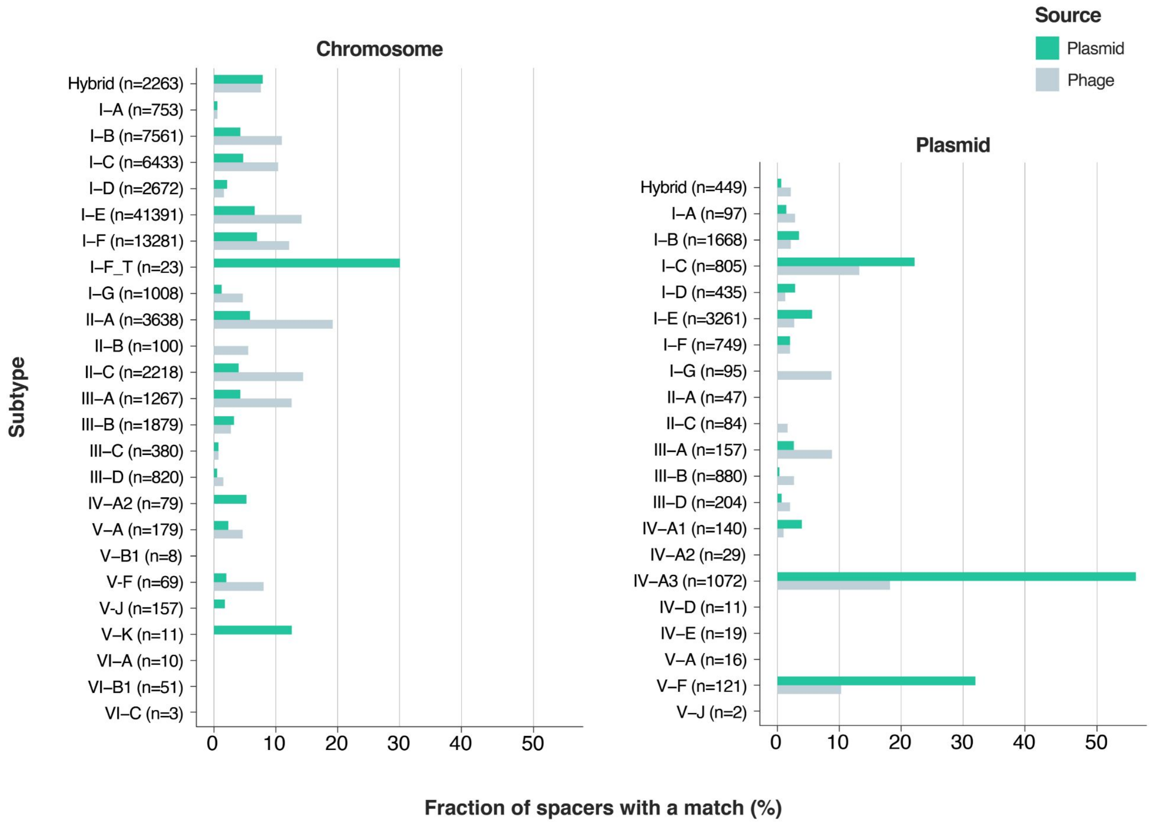

Source
Plasmid
Phage
Chromosome
Plasmid
Subtype
Hybrid (n=2263)
I–A (n=753)
I–B (n=7561)
I–C (n=6433)
I–D (n=2672)
I–E (n=41391)
I–F (n=13281)
I–F_T (n=23)
I–G (n=1008)
II–A (n=3638)
II–B (n=100)
II–C (n=2218)
III–A (n=1267)
III–B (n=1879)
III–C (n=380)
III–D (n=820)
IV–A2 (n=79)
V–A (n=179)
V–B1 (n=8)
V-F (n=69)
V-J (n=157)
V–K (n=11)
VI–A (n=10)
VI–B1 (n=51)
VI–C (n=3)
Hybrid (n=449)
I–A (n=97)
I–B (n=1668)
I–C (n=805)
I–D (n=435)
I–E (n=3261)
I–F (n=749)
I–G (n=95)
II–A (n=47)
II–C (n=84)
III–A (n=157)
III–B (n=880)
III–D (n=204)
IV–A1 (n=140)
IV–A2 (n=29)
IV–A3 (n=1072)
IV–D (n=11)
IV–E (n=19)
V–A (n=16)
V–F (n=121)
V–J (n=2)
0
10
20
30
40
50
Fraction of spacers with a match (%)

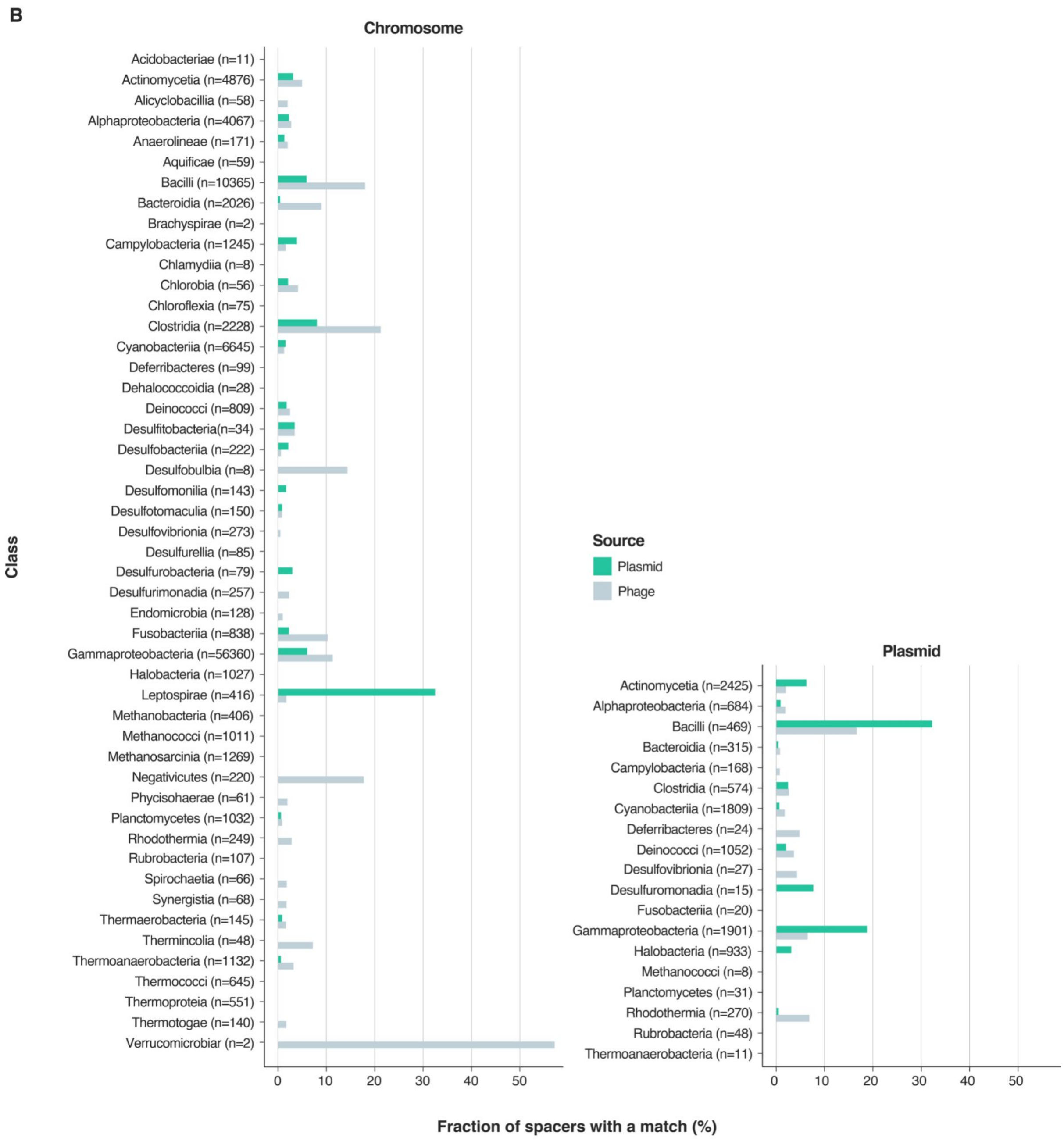

Supplementary Figure S13. Fractions of unique bacterial spacer-protospacer matches per total number of spacers for each individual subtype and host class. A) Chromosomal and plasmid spacer-match distribution broken down by CRISPR-Cas subtype. **B)** Chromosome- and plasmid-derived spacer-match distribution broken down by host taxonomy (class level). Numbers (n=X) indicate the number of unique spacers.

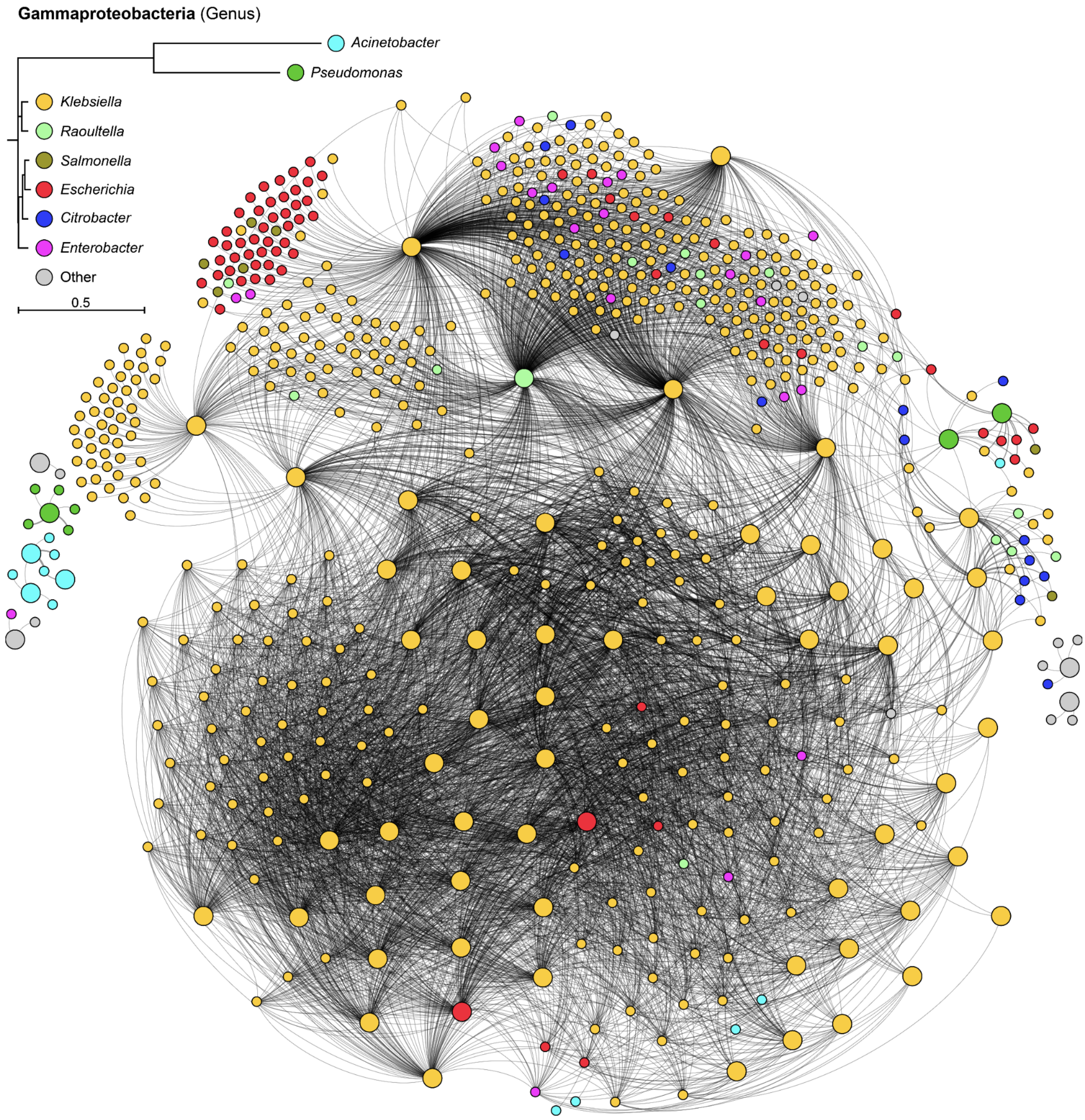

Supplementary Figure S14. Clustering network of predicted plasmid-plasmid CRISPR-Cas targeting in Gammaproteobacteria. The plasmid-plasmid targeting network is colored at the host genus level, where nodes correspond to individual plasmids and edges represent predicted spacer-protospacer matches. The phylogeny in the legend is based on the median cophenetic distance from the GTDB whole-genome phylogeny, with the tree inferred by neighbor-joining. "Other" indicates plasmids without a known host, a host with a different taxonomy than those displayed, or with a host with unspecific taxonomy. Large and small nodes indicate the presence or absence of CRISPR-Cas in the plasmid, respectively. Edge thickness is proportional to the number of spacer-protospacer matches between plasmid pairs.

Supplementary Datasheet S1. Compilation of detected CRISPR-Cas loci (on plasmids and plasmid-associated host chromosomes) and related information. Analysis of the data presented in Figure 5.

Supplementary Datasheet S2. Summary of the spacer-protospacer matches identified for plasmid and associated host chromosome-derived CRISPR array spacer contents. (Separate spreadsheet file).
